# Intraventricular Thrombosis After Myocardial Infarction: Prognostic Evaluation in Relation to Microvascular Obstruction Extent by CMR

**DOI:** 10.3390/jcm14082658

**Published:** 2025-04-13

**Authors:** Antonella Cecchetto, Francesco Zupa, Manuel De Lazzari, Angiola Bolis, Anna Baritussio, Stefano Nistri, Giorgio De Conti, Martina Perazzolo Marra

**Affiliations:** 1 Cardiology Unit, University of Padua-Azienda Ospedaliera, 35128 Padua, Italy; 2 Department of Cardio-Thoraco-Vascular Sciences and Public Health, University of Padua, 35128 Padua, Italy; 3 Unit of Cardiac Rehabilitation, ULSS 6 Euganea, 35020 Padua, Italy; 4 Cardiology Service, CMSR Veneto Medica, 36077 Altavilla Vicentina, Italy; 5 Radiology Unit, University of Padua-Azienda Ospedaliera, 35128 Padua, Italy

**Keywords:** anticoagulation, intraventricular thrombosis, microvascular obstruction, left ventricular remodeling

## Abstract

(1) **Background**: There are few data on anticoagulation therapy for left ventricular (LV) thrombosis following ST-segment elevation myocardial infarction (STEMI). The aim of this study was to assess whether microvascular obstruction (MVO) extent on cardiac magnetic resonance (CMR) worsened the prognosis of patients with LV thrombosis receiving anticoagulation. (2) **Methods**: reperfused STEMI patients undergoing CMR were enrolled. Patients were divided into 4 groups according to MVO and LV thrombosis presence or absence. Occurrence of major adverse cardiac events (MACE) was evaluated during follow-up. (3) **Results**: 80 STEMI patients were enrolled. According to MVO and LV thrombosis, 4 subgroups were obtained: patients with MVO and LV thrombosis (21 patients, 26%); patients with MVO without LV thrombosis (28 patients, 35%); patients without MVO with LV thrombosis (6 patients, 8%); patients without MVO and LV thrombosis (25 patients, 31%). All patients with LV thrombosis were treated with anticoagulation therapy. The median time to the follow-up was 11 months. Twenty-two patients (27%) experienced MACE. LV thrombosis treated with anticoagulation was an independent predictor of MACE (hazard ratio, 2.828; 95% confidence interval, 1.205–6.638; *p* = 0.017) and was associated with a worse prognosis (*p* = 0.012), regardless of MVO (*p* = 0.852), at Kaplan–Meier. (4) **Conclusions**: Patients with LV thrombosis treated with anticoagulation after a reperfused STEMI have a worse prognosis than those without; however, MVO extent did not worsen prognosis.

## 1. Introduction

Acute ST-segment elevation myocardial infarction (STEMI) can be complicated by left ventricular (LV) thrombosis. The occurrence of LV thrombi was found to be 12.3% among STEMI patients and 23.6% among anterior STEMI patients, as assessed by cardiac magnetic resonance (CMR) evaluation [1]. LV thrombosis was associated with larger infarct size (IS) and microvascular obstruction (MVO), reduced myocardial salvage, lower ejection fraction (EF), and an increased incidence of major adverse cardiac events (MACEs) [2]. MVO is found in up to 84% of patients after STEMI [3]. MVO develops when reperfusion is achieved following prolonged myocardial ischemia. The primary cause of this phenomenon, also termed no-reflow, is severe microvascular dysfunction or loss of integrity [4,5,6]. Intramyocardial hemorrhage is regarded as a severe form of MVO and follows the development of MVO within the core of the infarct. The causative factors include damage to the vascular endothelium and the accumulation of red blood cells in the myocardial extracellular space [7]. MVO has significant implications for successful restoration of post-ischemic tissue perfusion after coronary revascularization and, later, for possible adverse LV remodeling and heart failure [8,9,10]. In addition, MVO seems to be an important predictor of cardiac death, because patients with no-reflow more often had malignant arrhythmias and cardiac tamponade compared with patients without no-reflow [11]. One explanation for these findings may be the decreased wall thickness in MVO segments, which could result in increased wall stress [12].

Guidelines recommend anticoagulation for up to six months, guided by repeated imaging in patients with LV thrombosis (class of recommendation IIA, level of evidence C) [13,14]. It has to be considered that patients with STEMI undergoing PCI with stent implantation need dual antiplatelet therapy. Consequently, STEMI patients with LV thrombus exhibit a significantly increased risk of bleeding [15,16]. The presence of MVO could therefore lead to undertreatment of STEMI patients with concomitant LV thrombosis, based on the hypothesis that anticoagulation might increase the risk of myocardial rupture in these patients. Cardiac rupture is a fatal mechanical complication of acute myocardial infarction, and it has pathological characteristics similar to major bleeding: transmural infarct is associated with focal hemorrhage, neutrophilic infiltration, and lysis of myofibrils [17].

The effect of prolonged anticoagulation for LV thrombosis after STEMI with respect to MVO extent has not been clarified. The aim of this study was to assess whether MVO extent on CMR worsened the prognosis of patients anticoagulated for LV thrombosis.

## 2. Materials and Methods

### 2.1. Patients and Study Design

This was an observational, retrospective single-center cohort study. The study was conducted in accordance with the Declaration of Helsinki [18] and approved by the Ethics Committee on Human Research of Padua University (protocol code no. 20009, 20 March 2023). Because of the retrospective observational nature of the study, written consent was not required.

Consecutive STEMI patients treated with primary PCI who underwent CMR in the early post-infarction phase (within 7 days from symptom onset) at Padua University Hospital between April 2011 and January 2021 were evaluated for study inclusion. CMR indications were in line with clinical ethics practice. Coronary intervention and periprocedural treatment followed the European Society of Cardiology (ESC) guidelines [13]. Specifically, patients underwent primary PCI within 12 h from symptom onset. We excluded patients with previous cardiac events, any significant valvular diseases, cardiomyopathies, and other indications for anticoagulation without clinical documentation at admission and follow-up. Clinical variables (including age, gender, date of admission, risk factors for cardiovascular diseases, renal function by eGFR, site of myocardial infarction, pharmacological treatment with specific reference to antiplatelet and anticoagulation therapy) and echocardiographic data relative to hospitalization were collected using electronic archives. In each CMR exam, the presence of LV thrombi and MVO was verified. Patients were divided into 4 groups according to the presence or absence of MVO and LV thrombosis, as detected by CMR, in patients treated with prolonged anticoagulation therapy.

Follow-up was conducted at the Unit of Cardiac Rehabilitation of Padua. All patients underwent detailed assessment of medical history and complete transthoracic echocardiography between 3 and 12 months after STEMI. MACE and echocardiographic data were retrospectively collected from medical records. MACE was defined as reinfarction, congestive heart failure, cardiac death, coronary revascularization, re-hospitalization for angina pectoris, ventricular arrhythmias, stroke/transient cerebral ischemic events, recurrent intraventricular thrombosis and major bleeding requiring hospitalization.

### 2.2. CMR Acquisition Protocol and Analysis

All patients were studied during hospitalization, from day 1 to day 7 after admission, using a 1.5 T scanner (Magnetom Avanto; Siemens Healthineers, Erlangen, Germany). All images were acquired through dedicated cardiac software, phased-array surface receiver coil, and electrocardiogram triggering. All patients underwent a dedicated study protocol for acute myocardial infarction. The protocol included cine images for functional analysis, acquired in the long and short axis by applying steady-state free precession sequences (long axis: repetition time (TR), 3.5 ms; echo time (TE), 1.2 ms; and short axis: TR, 6.0 ms; TE, 1.0 ms; slice thickness, 7 mm; gap, 3 mm). Subsequently, breath-hold, black-blood, T2-weighted triple inversion–recovery sequences (TR, 2 xRR; TE, 61 ms; inversion time (TI), 160 ms; slice thickness, 7 mm) were acquired in the same slice positions as cine CMR. Late gadolinium enhancement (LGE) images were obtained in the same slice positions as cine CMR, completely encompassing the LV by applying segmented T1-weighted inversion recovery sequences (breath-hold segmented protocol with 10 ms echo spacing, TE 5.0 ms, and slice thickness 7 mm), 8 to 15 min after administration of contrast media (0.20 mmol/kg dose of gadobutrol).

**Figure 1:**
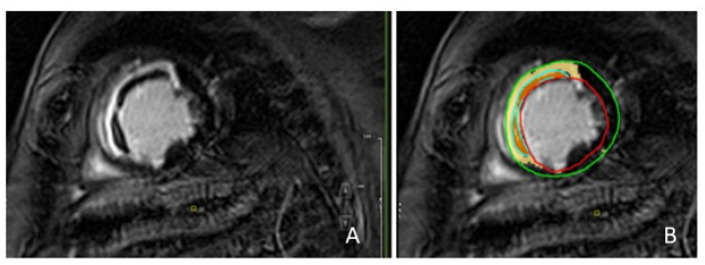
Patient with anterior and septal myocardial infarction. Late gadolinium images: infarcted myocardium was defined as hyper-enhanced myocardium; MVO was defined as a subendocardial dark area within the hyper-enhanced myocardium (**A**). Quantification of infarcted myocardium and MVO using a dedicated software: the yellow area identifies the infarcted myocardium; the orange area identifies MVO; the red line indicates the endocardial border; the green line indicates the epicardial border (**B**). MVO = microvascular obstruction.

Images were analyzed by consensus of two CMR-experienced operators (C.A. and Z.F.) blinded to clinical, laboratory, and angiographic findings. All images were analyzed using a dedicated software (CVI42, Circle Cardiovascular Imaging Inc; Calgary, Canada, Version 5.13.7). LV volumes, EF, and mass were calculated from the short-axis cine images by manually tracing the endocardial and epicardial borders. LV volumes and mass were normalized to body surface area. The presence, localization, and distribution patterns of edema and LGE were assessed visually on short- and long-axis images and defined as present only if detectable in two orthogonal planes. Infarcted myocardium, or IS, was measured from the short-axis LGE images with a threshold-based method (signal intensity > 5 standard deviation (SD) of unenhanced, remote myocardium). MVO was defined as a subendocardial dark area within the hyper-enhanced myocardium; it was assessed on LGE images and quantified by contouring the hypointense zone within the enhanced areas (Figure 1) using the “no reflow contour” application. IS and MVO were expressed as absolute amounts (grams) and normalized to the LV mass (% of LV). The presence of LV thrombi was looked for on early gadolinium enhancement (EGE) and/or LGE images with long TI (>440 msec).

### 2.3. Statistical Analysis

Each categorical variable is expressed as number and percentage of patients. Continuous data are reported as median with the corresponding interquartile range (IQR). Comparison of clinical, CMR, and echocardiographic baseline parameters between patients with or without MACE was performed with the chi-square test for categorical variables, and the Mann–Whitney U tests for continuous variables. We fitted univariable Cox regression analysis to estimate the predictors of MACE. We assessed survival curves using Kaplan–Meier distributions with log-rank comparison to illustrate the time-dependent occurrence of event-free survival in relation to the presence and extent of MVO and presence of LV thrombosis. To define the impact of the extent of MVO on outcome, Kaplan–Meier curves were generated considering the median value of MVO. A *p* value of less than 0.05 was considered statistically significant. Statistical analysis was performed using R software, version 4.1.0 222 (SPSS, Inc., Chicago, IL, USA).

## 3. Results

### 3.1. Population During Hospitalization

**Figure 2:**
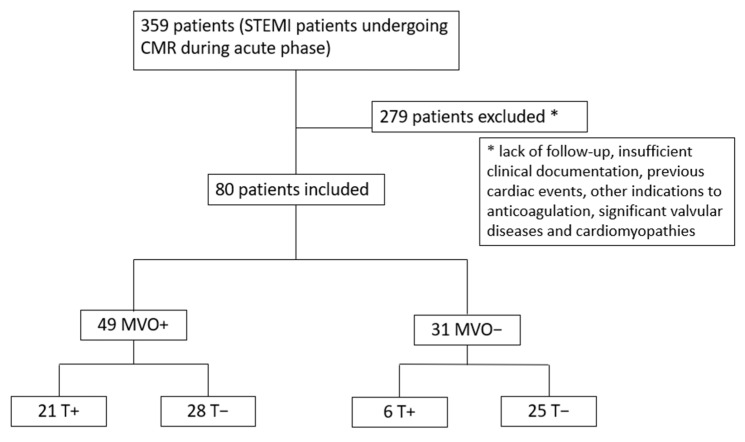
Study flow chart. MVO = microvascular obstruction; T = LV thrombosis; STEMI = ST-segment elevation myocardial infarction; CMR = cardiac magnetic resonance.

Among 359 STEMI patients undergoing CMR during the acute phase, 279 were lost during follow-up, did not have sufficient clinical documentation, or had previous cardiac events, other indications for anticoagulation, significant valvular disease, or a diagnosis of cardiomyopathy, leading to a final cohort of 80 patients [median age 67 years, IQR 57–76; 61 males (76%)]. After CMR evaluation, patients were categorized into two groups according to the presence [49 patients (61%)] or absence of MVO [31 patients (39%)]. Each group was divided into two subgroups according to the presence of LV thrombosis. Thus, 4 subgroups were obtained: group 1, patients with MVO and LV thrombosis [21 patients (26%)]; group 2, patients with MVO without LV thrombosis [28 patients (35%)]; group 3, patients without MVO with LV thrombosis [6 patients (8%)]; and group 4, patients without MVO and without LV thrombosis [25 patients (31%)] (Figure 2).

Patients with LV thrombus (twenty-seven patients, 34%) were discharged on prolonged anticoagulation therapy, of whom 23 were on warfarin and 4 were on low-molecular-weight heparin. The patients were started on anticoagulation therapy during hospital admission. The median duration of anticoagulation therapy was 6 months (IQR 4–9). Twenty-five patients were discharged with dual antiplatelet (clopidogrel and aspirin) and anticoagulation therapy, and 2 patients with single antiplatelet and anticoagulation therapy. Seventy-eight percent of anticoagulated patients had MVO at CMR. Seventy-four patients (93%) had at least one cardiovascular risk factor. Thirty-eight patients (48%) had anterior/septal or apical myocardial infarction. Baseline clinical characteristics are summarized in Table 1.

Baseline echocardiographic and CMR parameters are summarized in Table 2.

### 3.2. Predictors of Major Cardiovascular Events

The median duration of follow-up was 11 months (IQR 2–32). Twenty-two patients (27%) experienced MACE; in particular, 9% re-infarction, 17% congestive heart failure (including 3% cardiogenic shock), 4% re-hospitalization for angina, 1.3% cardiac death, 3% ischemic stroke/transient cerebral ischemia. No mechanical complications of STEMI were reported. No cases of recurrent intraventricular thrombosis and major bleeding events requiring hospitalization were documented. The median time between STEMI and adverse event was 4 months (IQR 2–9).

LVEF was measured with CMR, IS is expressed both as absolute mass and percentage of LV mass, eGFR, and LV thrombosis treated with anticoagulation therapy correlated with MACE; MVO mass did not correlate with MACE (Table 3).

Predictors of MACE during follow-up were obtained from univariate regression analyses. Data are shown in Table 4.

In the univariate logistic regression analysis, MACE was significantly associated with LVEF measured by CMR, LVESV measured by echocardiography, IS mass (absolute value and % of LV mass), eGFR, and LV thrombosis. Extent of IS > 18% of the LV mass (cut-off selected from the literature [19]), presence and extent of MVO > 2.19% (median value) of the LV mass were not predictors of MACE.

### 3.3. Event-Free Survival According to MVO and LV Thrombosis

**Figure 3:**
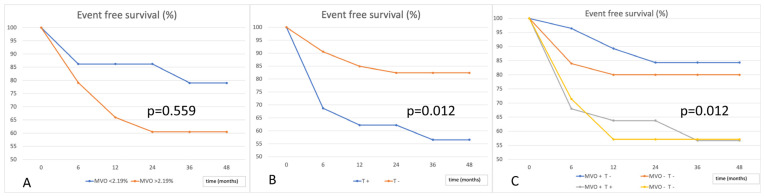
Event-free survival according to extent of MVO (**A**), presence of LV thrombosis (**B**), and the combination of MVO and LV thrombosis (**C**). T = LV thrombosis; MVO = microvascular obstruction.

We evaluated the outcome in terms of event-free survival according to the presence of MVO. Kaplan–Meier analysis did not reveal a significant correlation (*p* = 0.727). Considering the median value of MVO (expressed as % of LV mass) equal to 2.19%, to evaluate the relationship with the extent of MVO, Kaplan–Meier curves did not significantly differ (*p* = 0.559). With regards to the event-free survival, we found that intraventricular thrombosis was associated with a worse prognosis (*p* = 0.012). Combining the extent of MVO and LV thrombosis treated with anticoagulation therapy, Kaplan–Meier curves showed a clear distinction in terms of outcome depending on the presence of LV thrombosis (*p* = 0.012) (Figure 3).

## 4. Discussion

In this study, we investigated the prognostic impact of intraventricular LV thrombosis treated with anticoagulation therapy in reperfused STEMI patients, based on MVO presence on CMR. The main findings can be summarized as follows: (1) IS and LV thrombosis treated with anticoagulation were independent predictors of MACE in STEMI patients; (2) MVO extent on CMR did not worsen the prognosis of patients with LV thrombosis.

In our cohort we observed that the presence and extent of MVO did not predict MACE. MVO is not universally accepted as a prognostic factor, despite the majority of the studies associated MVO with LV remodeling and MACE [20,21]. There are, in fact, conflicting results with regards to the independent predictive role of MVO for MACE based on multivariate analysis studies [22,23,24,25,26,27]. De Waha et al. [9,28] in reperfused STEMI patients undergoing CMR at a median of 3 days after the index event, demonstrated that the presence and extent of MVO were independently associated with death, non-fatal myocardial re-infarction, and congestive heart failure. Similarly, Eitel et al., in a large, prospective, multicenter study, found that MVO ≥ 1.4% of LV emerged as an independent predictor of MACE [29]. Remarkably, MVO extent ≥ 2.6% of LV improved long-term risk stratification over traditional outcome predictors for Symons et al. [30]. However, recurrent cut-off of MVO extent on CMR predictive of MACE was not reported [31,32,33]. Few studies suggested, on the contrary, that MVO was not an independent predictor of outcome. In one of these studies, MVO was measured relatively early post-infarct (median of 4.5 h) which may have underestimated the incidence of MVO, since MVO likely increases in size over the first 24–48 h post-reperfusion [25]. In another study, results may have been influenced by heterogeneity in the times to epicardial revascularization, which affect the evolution of MVO [34]. The dynamic nature of MVO may, in effect, only provide a brief window on a pathophysiological process with long-term implications. Also, CMR assessment of MVO is limited by the lack of standardization with regards to how and when MVO is measured, both with respect to the time following contrast administration and the time post-reperfusion. Further explanations of our results could be the relatively small number of events at follow-up and the heterogeneity of outcomes included.

We described that IS expressed as % of LV mass was an independent predictor of MACE. However, based on a literature-derived cut-off for IS > 18% [19], a significant correlation with events was not confirmed. We could interpret this data considering the lack of a clear IS cut off for prognosis. IS can vary greatly at different time points after myocardial infarction, due to the amount of edema associated with necrosis [35]. In the literature, consequently, there is conflicting evidence to support its predictive value for MACE. The direct relationship between CMR-determined IS and the risk of developing cardiovascular complications after infarction was demonstrated, with larger infarcts correlating with worse clinical outcomes [19,36]. In multivariable analysis, infarct extent by LGE imaging but not its location was independently associated with an increased risk of MACE [37]. De Waha et al. [9] noted that IS, although predictive in the univariable analysis, did not remain significant in the multivariable model including MVO and established prognostic markers and scores. IS ≥ 25% of the LV mass, adjusted for MVO and LVEF, was not associated with MACE for van Kranenburg et al. [24]. Also, in this case, different results can be attributed to the follow-up duration, the sample size, the study design, the CMR protocol, the method used to quantify the IS, the imaging time points after infarction, the inclusion of unstable angina, or repeat revascularization in the combined clinical endpoint.

The effect of LV thrombosis on MACE after STEMI, according to presence of MVO, is not known. In our study, LV thrombosis was an independent predictor of MACE, with a strong association with respect to others factor. The reason why LV thrombosis treated with anticoagulation therapy influences prognosis without causing major bleeding can be sought in the greater complexity of patients with LV thrombosis: patients with intraventricular thrombosis could be those with more extensive IS, complex coronary artery disease, LV dysfunction and progressive remodeling, and comorbidities. In fact, intraventricular thrombosis is more prevalent among patients with anterior infarction, moderate LV dysfunction, and adverse LV remodeling [1]. However, MVO extent on CMR did not worsen the prognosis of patients anticoagulated for LV thrombosis, probably because the presence of MVO in the acute postinfarction period was associated with a greater rate of fibrous scar formation at follow-up. There is a linear relation between MVO extent and scar size, not only in the acute but also in the chronic setting, as MVO in the acute phase is a predictor of transmural extent at 6 months [19]. Accordingly, MVO should not influence therapeutic strategy (i.e., starting anticoagulation therapy) when necessary to resolve an intraventricular thrombosis. Our findings may become relevant for identifying patients at high risk who may benefit from closer follow-up.

There are several limitations in our study. A high percentage of patients undergoing CMR showed LV thrombosis, probably because only the more complex patients were studied, according to good clinical practice. One limitation was the small sample size and the unbalanced distribution of patients in the 4 groups. In particular, group 3 (patients without MVO on anticoagulation) was underrepresented; in fact, patients after STEMI with LV thrombosis frequently had MVO. The effects of anticoagulation therapy and intraventricular thrombosis are indistinguishable; it may be useful to use a comparison population undergoing anticoagulation therapy for other reasons. Further studies could evaluate the effect of anticoagulation only on intramyocardial hemorrhage. Patients presenting more than 12 h after symptom onset were excluded from the study. Furthermore, being a retrospective study, many patients did not have significant clinical and instrumental follow-up documentation.

## 5. Conclusions

Patients with LV thrombosis after a reperfused STEMI have a worse prognosis than those without; however, MVO extent, within the limits of our study, did not worsen the prognosis.

## Figures and Tables

**Table 1:** Baseline clinical characteristics of patients.

	Patients (*n* = 80)	MVO + T+ (*n* = 21)	MVO + T− (*n* = 28)	MVO − T+ (*n* = 6)	MVO − T– (*n* = 25)	*p*
CV risk factors, *n* (%)						
Hypertension	44 (55)	15 (71)	17 (61)	3 (5)	9 (36)	0.121
Dyslipidemia	60 (75)	20 (95)	20 (71)	4 (67)	16 (64)	0.345
Smoke	42 (53)	16 (57)	13 (46)	2 (33)	11 (44)	0.457
Diabetes	3 (4)	2 (10)	1 (4)	0	0	0.091
CAD family history	24 (30)	7 (33)	9 (32)	2 (33)	6 (24)	0.874
eGFR ml/min/1.73 m^2^ median IQR (range)	79 (67–92)	70 (67–89)	78 (75–92)	79 (70–85)	80 (67–93)	0.786
Culprit lesion, *n* (%)						
LAD	40 (50)	20 (95)	10 (36)	6 (100)	4 (16)	0.091
LCX	23 (28)	0	10 (36)	0	11 (44)	0.237
RCA	17 (21)	1 (5)	8 (29)	0	10 (40)	0.670

LCX: left circumflex coronary artery; AMI = acute myocardial infarction; CAD = coronary artery disease; CV = cardiovascular; eGFR = estimated glomerular filtration rate; IQR = interquartile range; LAD = left anterior descending coronary artery; MVO = microvascular obstruction; *n* = number; ns = not significant; RCA = right coronary artery; T = LV thrombosis.

**Table 2:** Baseline echocardiographic and CMR parameters of patients.

	Patients (*n* = 80) Median IQR (Range)	MVO + T+ (*n* = 21) Median IQR (Range)	MVO + T− (*n* = 28) Median IQR (Range)	MVO − T+ (*n* = 6) Median IQR (Range)	MVO − T− (*n* = 25) Median IQR (Range)	*p*
CMR parameters						
LVEDV ml/m^2^	94 (81–110)	94 (81–109)	94 (86–108)	108 (81–125)	94 (78–106)	0.789
LVESV ml/m^2^	56 (43–68)	56 (49–69)	59 (48–67)	50 (41–79)	46 (39–58)	0.896
EF%	43 (33–51)	40 (36–47)	40 (34–48)	41 (34–52)	47 (40–51)	0.444
IS mass g	39 (25–58)	46 (28–60)	47 (38–60)	23 (16–40)	24 (15–29)	0.676
IS mass/LV mass%	33 (23–42)	37 (27–44)	39 (29–46)	26 (14–36)	22 (14–32)	0.569
MVO mass g	0.53 (0.01–4.47)	3.25 (0.43–9.08)	1.84 (0.68–5.73)	0	0	0.095
MVO mass/LV mass %	0.45 (0.01–2.84)	2.58 (0.04–3.56)	1.46 (0.03–2.89)	0	0	0.085
Echo parameters						
LVEDV ml/m^2^	68 (56–81)	67 (56–79)	69 (60–79)	69 (59–93)	62 (52–72)	0.433
LVESV ml/m^2^	39 (29–49)	40 (33–47)	43 (32–50)	44 (29–59)	33 (28–44)	0.326
EF%	43 (35–49)	40 (36–47)	40 (35–48)	41 (33–51)	47 (42–52)	0.685

A = anticoagulation for LV thrombosis; CMR = cardiac magnetic resonance; EDV = end diastolic volume; EF = ejection fraction; ESV = end systolic volume; IQR = interquartile range; IS = infarct size; LV = left ventricle; MVO = microvascular obstruction; *n* = number; ns = not significant; T = LV thrombosis.

**Table 3:** Baseline characteristics distribution with regards to the presence or absence of major cardiovascular events during follow-up.

Baseline Characteristics	Absence of MACE *n* = 58	Presence of MACE *n* = 22	*p*
CMR LVEF% median IQR (range)	43 (35–53)	35 (30–48)	0.048
Echo LVEF% median IQR (range)	43 (36–49)	39 (29–50)	0.094
CMR LVEDV mL/m^2^ median IQR (range)	94 (82–109)	95 (79–127)	0.476
CMR LVESV mL/m^2^ median IQR (range)	56 (42–65)	59 (47–86)	0.276
Echo LVEDV mL/m^2^ median IQR (range)	68 (54–78)	66 (60–82)	0.443
Echo LVESV mL/m^2^ median IQR (range)	38 (28–46)	42 (32–59)	0.194
IS mass g median IQR (range)	34 (19–53)	45 (28–70)	0.027
IS mass (%LV) median IQR (range)	32 (17–40)	39 (28–53)	0.031
MVO mass g median IQR (range)	0.43 (0.01–3.14)	0.43 (0.01–5.51)	0.594
MVO mass (%LV) median IQR (range)	0.21 (0.01–2.22)	0.38 (0.01–3.82)	0.467
eGFR mL/min/1.73 m^2^ median IQR (range)	80 (68–91)	66 (52–89)	0.038
LV thrombus treated with anticoagulation (%)	30	59	0.016

CMR = cardiac magnetic resonance; EDV = end diastolic volume; EF = ejection fraction; eGFR = estimated glomerular filtration rate; ESV = end systolic volume; IQR = interquartile range; IS = infarct size; LV = left ventricle; MACE = major adverse cardiovascular event; MVO = microvascular obstruction; *n* = number.

**Table 4:** Predictors of major cardiovascular events.

Baseline Characteristics	Univariate Analysis		
	HR	CI	*p*
CMR LVEF%	0.96	0.93–0.99	0.032
Echo LVEF%	0.95	0.91–1.00	0.050
Echo LVEDV ml/m^2^	1.01	0.99–1.02	0.690
Echo LVESV ml/m^2^	1.02	1.00–1.04	0.032
IS mass g	1.02	1.00–1.04	0.013
IS mass (%LV)	1.04	1.01–1.07	0.008
IS mass > 18%	3.49	0.82–14.97	0.092
MVO presence	1.09	0.46–2.59	0.854
MVO mass	1.06	0.99–1.13	0.086
MVO mass (%LV)	1.09	0.99–1.21	0.079
MVO mass > 2.19%	1.64	0.70–3.83	0.257
eGFR	0.98	0.96–1.00	0.016
LV thrombus treated with anticoagulation	2.83	1.21–6.64	0.017

CMR = cardiac magnetic resonance; CI = confidence interval; EDV = end diastolic volume; EF = ejection fraction; eGFR = estimated glomerular filtration rate; HR = hazard ratio; ESV = end systolic volume; HR = hazard ratio; IS = infarct size; LV = left ventricle; MVO = microvascular obstruction.

## Data Availability

The data presented in this study are available upon request from the corresponding author. The data are not publicly available due to privacy reasons.
